# Mechanisms of aquaporin‐4 vesicular trafficking in mammalian cells

**DOI:** 10.1111/jnc.16029

**Published:** 2023-12-16

**Authors:** Andrea Markou, Philip Kitchen, Ahmed Aldabbagh, Mariaelena Repici, Mootaz M. Salman, Roslyn M. Bill, Zita Balklava

**Affiliations:** ^1^ College of Health and Life Sciences Aston University Birmingham UK; ^2^ School of Biosciences, Faculty of Health and Medical Sciences University of Surrey Guildford UK; ^3^ Department of Physiology, Anatomy and Genetics University of Oxford Oxford UK; ^4^ Kavli Institute for NanoScience Discovery University of Oxford Oxford UK

**Keywords:** aquaporin‐4, astrocyte, cytoskeleton, oedema, Rab GTPase, vesicular trafficking

## Abstract

The aquaporin‐4 (AQP4) water channel is abundantly expressed in the glial cells of the central nervous system and facilitates brain swelling following diverse insults, such as traumatic injury or stroke. Lack of specific and therapeutic AQP4 inhibitors highlights the need to explore alternative routes to control the water permeability of glial cell membranes. The cell surface abundance of AQP4 in mammalian cells fluctuates rapidly in response to changes in oxygen levels and tonicity, suggesting a role for vesicular trafficking in its translocation to and from the cell surface. However, the molecular mechanisms of AQP4 trafficking are not fully elucidated. In this work, early and recycling endosomes were investigated as likely candidates of rapid AQP4 translocation together with changes in cytoskeletal dynamics. In transiently transfected HEK293 cells a significant amount of AQP‐eGFP colocalised with mCherry‐Rab5‐positive early endosomes and mCherry‐Rab11‐positive recycling endosomes. When exposed to hypotonic conditions, AQP4‐eGFP rapidly translocated from intracellular vesicles to the cell surface. Co‐expression of dominant negative forms of the mCherry‐Rab5 and ‐Rab11 with AQP4‐eGFP prevented hypotonicity‐induced AQP4‐eGFP trafficking and led to concentration at the cell surface or intracellular vesicles respectively. Use of endocytosis inhibiting drugs indicated that AQP4 internalisation was dynamin‐dependent. Cytoskeleton dynamics‐modifying drugs also affected AQP4 translocation to and from the cell surface. AQP4 trafficking mechanisms were validated in primary human astrocytes, which express high levels of endogenous AQP4. The results highlight the role of early and recycling endosomes and cytoskeletal dynamics in AQP4 translocation in response to hypotonic and hypoxic stress and suggest continuous cycling of AQP4 between intracellular vesicles and the cell surface under physiological conditions.

## Graphical abstract

Lack of specific and therapeutic pore‐blocking AQP4 inhibitors highlights the need to explore alternative routes to control the water permeability in glial cell membranes that facilitate brain swelling following insults, such as traumatic injury or stroke. In this study, we explored molecular mechanisms of AQP4 trafficking in mammalian cells. AQP4 continuously cycles between the cell surface, Rab5‐positive early and Rab11‐positive recycling endosomes in mammalian cells. AQP4 internalisation is dynamin‐dependent and AQP4 translocation mechanisms are impaired upon inhibition of Rab5 and Rab11 function as well as cytoskeleton dynamics revealing potential targets for oedema treatment. Created with biorender.com.

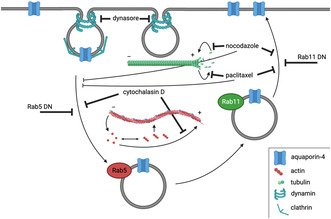

**Abbreviations**
AM: astrocyte medium
ANOVA: analysis of variance
AQP: aquaporin
CaM: calmodulin
cAMP: cyclic adenosine monophosphate
CIE: clathrin‐independent endocytosis
CKII: casein kinase II
CME: clathrin‐mediated endocytosis
CNS: central nervous system
DMEM: Dulbecco's modified Eagle's medium
eGFP: enhanced green fluorescent protein
FBS: fetal bovine serum
FDA: food and drug administration
HEK293: human embryonic kidney 293 cells
MDCK: Madin‐Darby canine kidney cells
PBS: phosphate buffered saline
PCC: Pearson's correlation coefficient
PEI: polyethylenimine
PKA: protein kinase A
Rab: Ras‐associated binding protein
RME: relative membrane expression
RRID: Research Resource Identifier
TRPV4: transient receptor potential vanilloid 4

## 1 INTRODUCTION

Aquaporin (AQP) water channels are transmembrane proteins facilitating bidirectional transport of water and selected small polar solutes across biological membranes. AQPs are present in all organisms and in humans 13 AQPs have been identified with distinct tissue expression (Markou et al., 2022). AQP4 is highly expressed in astrocytes in the central nervous system (CNS) where it plays a crucial role in water balance, astrocyte migration, neuroexcitation, neuroinflammation and the proper functioning of the glymphatic system (Fukuda & Badaut, 2012; Jung et al., 1994; Markou et al., 2022; Saadoun et al., 2005; Salman et al., 2021). AQP4 is also present in the collecting duct and parts of proximal tubule of the kidney where it plays a role in water reabsorption (Kim et al., 2001). The expression of AQP4 in the brain is predominantly in areas close to fluid‐filled compartments, indicating that AQP4 plays a role in brain fluid homeostasis (Salman, Kitchen, Halsey, et al., 2022). Two other family members, AQP1 and AQP9, are also expressed in the brain, AQP1 in the choroid plexus under normal conditions and both AQP1 and AQP9 in astrocytes in response to various stresses (Badaut et al., 2002). AQP4 is abundant in the subpial processes forming the interface between the central nervous system (CNS) and cerebrospinal fluid and at the endfeet, forming part of the interface between the blood and CNS (Papadopoulos & Verkman, 2013). The osmotic or hydrostatic pressure gradient across the cell membrane determines the flow of water through AQP4 channels, with the flow rate depending on the number of AQP4 channels present.

As the most abundantly expressed water channel in the CNS, AQP4 plays a role in cerebral oedema (Vandebroek & Yasui, 2020). Following a trauma to the brain, ischaemia, inflammation or a tumour, influx of water into astrocytes occurs via AQP4 channels, associated with an increase in volume of the brain tissue, cytotoxic oedema and increased intracranial pressure which has detrimental effects in terms of morbidity and mortality. Understanding the molecular mechanisms that lead to brain swelling is vital in order to identify and develop effective and targeted treatments. Although AQPs have been validated as drug targets for many years, it remains unclear whether their pores are intrinsically undruggable and whether small drug‐like molecules can be identified that block them (Salman, Kitchen, Yool, & Bill, 2022). Recently, AQP4 subcellular trafficking has been validated as a potential target for oedema treatment. This was done through the administration of the Food and Drug Administration (FDA) licenced trifluoperazine to rats subjected to spinal crush, traumatic brain injury and stroke, leading to a reduction in oedema and enhanced functional and behavioural recovery (Kitchen et al., 2020; Sylvain et al., 2021). This study established the subcellular relocalisation mechanism of AQP4 as an attractive therapeutic target.

The translocation mechanisms of the 13 human AQPs have been described to varying degrees (Markou et al., 2022). The most well understood is that of AQP2, which is expressed in the kidney collecting duct and is triggered by vasopressin (Centrone et al., 2022). The trafficking mechanism of AQP4 has been shown to be calmodulin (CaM) and phosphorylation dependent (Ishida et al., 2022; Kitchen, Day, Salman, et al., 2015; Kitchen, Day, Taylor, et al., 2015). Triggered by changes to the extracellular environment, Ca^2+^ ions enter the cell via TRPV4 channels and activate calmodulin. Once activated, calmodulin binds directly to AQP4, as well as activating protein kinase A (PKA) via an adenylyl cyclase which leads to phosphorylation of AQP4 and its subsequent translocation to the plasma membrane (Kitchen et al., 2020). The phosphorylation that occurs following PKA activation most likely occurs on endosomal membranes. However, following this phosphorylation, there is little known about the membrane trafficking of the phosphorylated AQP4. Studies in the HGT1 human gastric cancer cell line have revealed that histamine treatment induced internalisation and subsequent phosphorylation of transfected AQP4 by PKA. AQP4 phosphorylation was crucial for AQP4 return to the cell surface following histamine washout (Carmosino et al., 2007). In human embryonic kidney 293 cells (HEK293) and rat primary astrocytes, AQP4 internalisation and translocation was shown to depend on phosphorylation at S276 by PKA (Kitchen, Day, Salman, et al., 2015). Phosphorylation of S276, along with S285, T289 and S316 by casein kinase II (CKII) in mouse primary astrocytes was shown to target AQP4 to the Golgi, and mutations at these locations prevented mouse AQP4 from translocating to the cell surface (Kadohira et al., 2008).

Within the cytoplasm, AQP4 has been shown to be present in vesicles (Mazzaferri et al., 2013). Analysis of their movement indicated that the distance of vesicles from the plasma membrane did not affect their behaviour and the rate of AQP4‐positive vesicle movement towards and away from the plasma membrane was similar (Mazzaferri et al., 2013). In rat cortical astrocytes AQP4 M1, M23 and the AQP4e isoform (which does not exist in human) have been demonstrated to localise to the plasma membrane and play a role in cell volume regulation. AQP4b and AQP4d isoforms have been shown to localise with early endosomes, late endosomes and Golgi and AQP4d colocalization with early endosomes was enhanced following hypoosmotic stress; however, these isoforms are missing two transmembrane helices and whether they function as water channels is unclear (Lisjak et al., 2020). In HEK293 cells and rat primary cortical astrocytes AQP4‐carrying vesicles have been shown to translocate to the cell surface in response to changes in tonicity, hypoxia and hypothermia (Kitchen et al., 2020; Kitchen, Day, Taylor, et al., 2015; Salman et al., 2017) and require cytoskeleton support (Mazzaferri et al., 2013; Potokar et al., 2013). In rat astrocytes, internalisation of AQP4 to the early endosome pool is inhibited by laminin treatment, and further regulated by dystroglycan and dynamin (Tham et al., 2016). It remains an open question how AQP4‐containing vesicles translocate to and from the cell surface.

We hypothesised that AQP4 translocation between the cell surface and intracellular compartments utilises early and recycling endosomes and depends on cytoskeletal dynamics. Major regulators of vesicular transport are Ras‐associated binding (Rab) proteins. Vesicular trafficking along short and long distances depends on cytoskeletal dynamics. Additionally, we explored whether AQP4 internalisation occurs via clathrin‐dependent or independent routes.

## 2 MATERIALS AND METHODS

### 2.1 Materials

*Plasmids*: mCherry‐Rab5a‐7 (RRID:Addgene_55126) and mCherry‐Rab11a‐7 plasmids (RRID:Addgene_55124) were a gift from Michael Davidson. DsRed‐rab11DN(S25N) was a gift from Richard Pagano (RRID:Addgene_12680); mCherry‐Rab5DN(S34N) was a gift from Sergio Grinstein (RRID:Addgene_35139); Human AQP4 was cloned into mammalian expression vector pDEST47 (Invitrogen, Cat. nr. 12281010). *Antibodies*: Anti‐AQP4 antibody (RRID:AB_11143780); HRP‐conjugated secondary antibody (RRID:AB_2099233); anti‐GFAP (RRID:AB_1209224); Anti‐EAAT1 (RRID:AB_10695722); Anti‐S100β (RRID:AB_2665776); and Alexa Fluor 488 secondary antibodies (RRID:AB_143165, AB_2534069).

### 2.2 Cell culture

Cell lines used in this study were human embryonic kidney 293 (HEK293) (RRID:CVCL_0045) and primary human astrocytes (ScienCell, USA, Cat. Nr. #1800). None of the cell lines are listed as a commonly misidentified cell line by the International Cell Line Authentication Committee (ICLAC; http://iclac.org/databases/cross‐contaminations/). HEK293 were used between passages 3 and 14 and were cultured in Dulbecco's modified Eagle's medium (DMEM) supplemented with 10% fetal bovine serum (FBS), penicillin (100 units/mL) and streptomycin (100 μg/mL) and L‐glutamine (6 mM) at 37°C with 5% CO_2_ (HEK293 identity was confirmed by ATCC). Human cortical astrocytes were used for experiments from passages 2–5 and regularly checked for mycoplasma infection. Astrocyte identity was confirmed by immunofluorescence for GFAP, EAAT1 and S100β (Figure S1). Cells were cultured in astrocyte medium (AM) (ScienCell, USA), supplemented with 2% FBS, 1% astrocyte growth supplement and 1% Penicillin/streptomycin solution. Cells were grown in a 37°C incubator with 5% CO_2_. When using hypoxia as a trigger, cells were incubated in a hypoxic chamber under 1% O_2_ and 5% CO_2_. All experiments were performed in vitro, and institutional ethics approval was not required for this study. All experimental work was carried out at Aston University.

### 2.3 Transient transfection with polyethylenimine (PEI)

A mixture containing 50 μL serum‐free DMEM, 1 μg plasmid DNA and 6 μL of 1 mg/mL PEI in dH_2_O was vortexed for 15 s and incubated at room temperature for 10 min. This was added to 300 μL of DMEM with serum. Cell culture medium was removed from the plates and the transfection mixture was pipetted dropwise onto the cells grown in 35 mm dishes. The cells were incubated for 3 h, then 2 mL DMEM with serum was added and the plates were returned to the incubator at 37°C for 24 h.

### 2.4 Confocal microscopy

For colocalisation studies, HEK293 cells were grown on coverslips in 6‐well plates and transfected with the plasmids when they had reached 60% confluency. After 24 h, the coverslips were mounted on slides using Mowiol and were then imaged on a Leica SP8 FALCON Leica confocal microscope equipped with white light laser with tunable excitation from 470 to 670 nm. Slides were imaged in sequential mode using a HC PL APO 63× 1.4 NA oil immersion lens. The following settings were used: Alexa 488—excitation 499 nm laser line, emission detected between 504 and 568 nm; Alexa 594—excitation 594 nm laser line, emission detected between 600 and 650 nm. Experiments were repeated three times and, in each experiment, colocalisation was quantified for 10 cells.

### 2.5 Live imaging of AQP4‐eGFP translocation

Cells were grown in 35 mm FluoroDishes (World Precision Instruments) and transfected with the plasmids when they had reached 60% confluency. The live cells were placed on a temperature‐controlled stage on a Zeiss Axiovert 200 M fluorescence (Carl Zeiss) microscope and imaged with a Hamamatsu Orca camera controlled by Volocity software (Improvision) using 63× 1.4 NA oil immersion lens after 90 s. Following this, a 3:1 mixture of water: DMEM replaced the DMEM and images were taken after 5 min. Finally, the hypotonic solution was replaced with DMEM and cells were imaged again after 5 min. In experiments where AQP4 trafficking was measured in the presence of compounds, the following concentrations and pre‐incubation times were used: dynasore (Abcam, Cat. nr. ab120192) 10 μM, 30 min (Bandmann et al., 2019); filipin (Sigma Aldrich, Cat. nr. F9765) 15 μM, 45 min (Orlandi & Fishman, 1998); jasplakinolide (Abcam, Cat. nr. ab141409) 150 nM, 30 min (Lalo et al., 2011); paclitaxel (Abcam, Cat. nr. ab120143) 50 nM, 20 min (Liebmann et al., 1993); nocodazole (Abcam, Cat. nr. ab120630) 30 μM, 45 min (Yea et al., 2007); cytochalasin D (Abcam, Cat. nr. ab143484) 5 μM, 45 min (Haghparast et al., 2015); dyngo‐4a (Abcam, Cat. nr. ab120689) 30 μM, 30 min (Harper et al., 2011); and pitstop 2 (Sigma Aldrich, Cat. nr. SML1169), 20 μM, 45 min (Dutta et al., 2012); These were determined based on referenced literature searches to establish optimal timepoints and concentrations.

### 2.6 Colocalisation analysis

Two methods of analysis were used to determine co‐localisation: Pearson's correlation coefficient (PCC) and Mander's colocalisation coefficients, *M*_1_ and *M*_2_. The JaCoP plugin in ImageJ was used to analyse the images and calculate the two coefficients. The following formulae were applied to calculate PCC and M1 and M2 respectively:

$$\begin{array}{c} r_{p}\frac{\sum_{i}\left(A_{i}-a\right)\times \left(B_{i}–b\right)}{\sqrt{\sum_{i}{\left(A_{i}-a\right)}^{2}\times \sum_{i}{\left(B_{i}-b\right)}^{2}}}\begin{array}{c} M_{1}=\frac{\sum_{i}A_{i,\text{coloc}}}{\sum A_{i}}\;\text{with}\;A_{i,\text{coloc}}=A_{i},\text{if}\;B_{i}>0\;\\ M_{2}=\frac{\sum_{i}B_{i,\text{coloc}}}{\sum B_{o}}\;\text{with}\;B_{i,\text{coloc}}=B_{i},\text{if}\;A_{i}>0 \end{array}. \end{array}$$

The PCC gives an indication of whether the two proteins are associated within the same area and the strength of this association. A value of −1 shows no correlation and that the two proteins are present in the completely opposite space; a value of 0 shows no correlation and the two proteins are present in random spaces and a value of +1 shows perfect correlation, with the two proteins present in the same space. The M1 and M2 coefficients give an indication of co‐occurrence, or, in other words, colocalisation. This is calculated by adding the fluorescence intensity of the pixels of the two channels and then dividing that by the integrated density of the pixels. A value of 0 shows that there is no colocalisation and a value of 1 shows there is complete colocalisation. This method measures only the pixels that are covered by both signals.

### 2.7 Relative membrane expression

Live cell images were analysed in ImageJ through the measurement of intensity profiles across each cell. Relative membrane expression (RME) was calculated as previously described (Conner et al., 2010). Each experimental repeat consisted of three profiles per cell of at least 10 cells per condition in each repeat.

### 2.8 Cell surface biotinylation

Cell surface biotinylation was done as described previously (Cho & Roche, 2019; Kitchen, Day, Salman, et al., 2015; Tham et al., 2016). Briefly, human astrocytes were plated in 48‐well plates 24 h before the experiment. The cells were placed in astrocyte medium or astrocyte medium diluted with dH_2_O (in a ratio of 1:3) with or without different inhibitors. Following incubation, the plates were placed on ice and each well was washed five times by removing 375 μL of the media and replacing with 375 μL ice‐cold phosphate‐buffered saline (PBS) or PBS diluted to the same tonicity as the DMEM. The cells were then incubated on ice for 30 min with 600 μL of EZ‐Link Sulfo‐NHS‐SS‐Biotin (ThermoFisher, Cat nr. A39258) in PBS at the same tonicity as in previous steps. If internalisation was measured, the cells were incubated at 37°C for 30 min following a wash with 500 μL PBS and replacement of fresh 500 μL media. Then, cells were placed back on ice, washed three times with PBS and reduced for 15 min with 500 μL of glutathione solution (50 mM glutathione, 75 mM NaCl and 7 mM NaOH) and the reduced cysteine residues were quenched with 50 mM iodoacetamide in PBS three times for 5 min. Following this, the quenching solution was the same for all biotinylation assays (25 mM glycine in PBS) and was added three times for 5 min. The cells were then lysed on ice for 45 min in Tris‐Triton lysis buffer (1% v/v Triton X‐100, 100 mM NaCl, 2 mM MgCl_2_ and 25 mM Tris pH 7.4). Cell lysates were transferred to 1.5 mL Eppendorf tubes and centrifuged at 4°C for 10 min at 20 000 *g* and then 100 μL of supernatant were added to neutravidin‐coated high binding capacity 96‐well plates (ThermoFisher, Cat nr. 15510) and were incubated at 4°C for 2 h. The plates were then washed with 100 μL 0.05% (v/v) PBS‐Tween and blocked for 1 h in 100 μL 3% (w/v) BSA in PBS at room temperature on an orbital shaker. Following this, plates were incubated with 100 μL AQP4 antibody diluted 1:2000 in 0.05% (v/v) PBS‐Tween overnight at 4°C. The plates were then washed with 100 μL 0.05% (v/v) PBS‐Tween and incubated with 100 μL HRP‐conjugated secondary antibody diluted 1:2500 for 1 h at room temperature. Finally, the plates were washed with 100 μL 0.05% (v/v) PBS‐Tween three times for 5 min and incubated with 100 μL OPD HRP substrate (Sigma Aldrich, Cat. nr. P9187) for 45 min at room temperature, wrapped in foil to avoid exposure to light. Absorbance was read at 450 nm.

### 2.9 Image and statistical analysis

Data were assessed for normality, using the Shapiro–Wilk test. All data showed a *p* > 0.05 for this, indicating they were normally distributed. The generalised ESD test was conducted to determine whether significant outliers existed. The test showed no outliers, and no data points were excluded from the analysis.

Colocalisation and live AQP4‐eGFP image analysis was done using FIJI (NIH, USA); statistical analysis was done using GraphPad Prism 6.01 (GraphPad Software Inc., USA) using a one‐way analysis of variance (ANOVA) multiple comparison with Tukey's post hoc test. A *p* < 0.05 was used as a threshold for results that were considered to be statistically significant. Actual p values are listed in Tables S1–S14.

## 3 RESULTS

### 3.1 AQP4‐eGFP uptake routes

**FIGURE 1:**
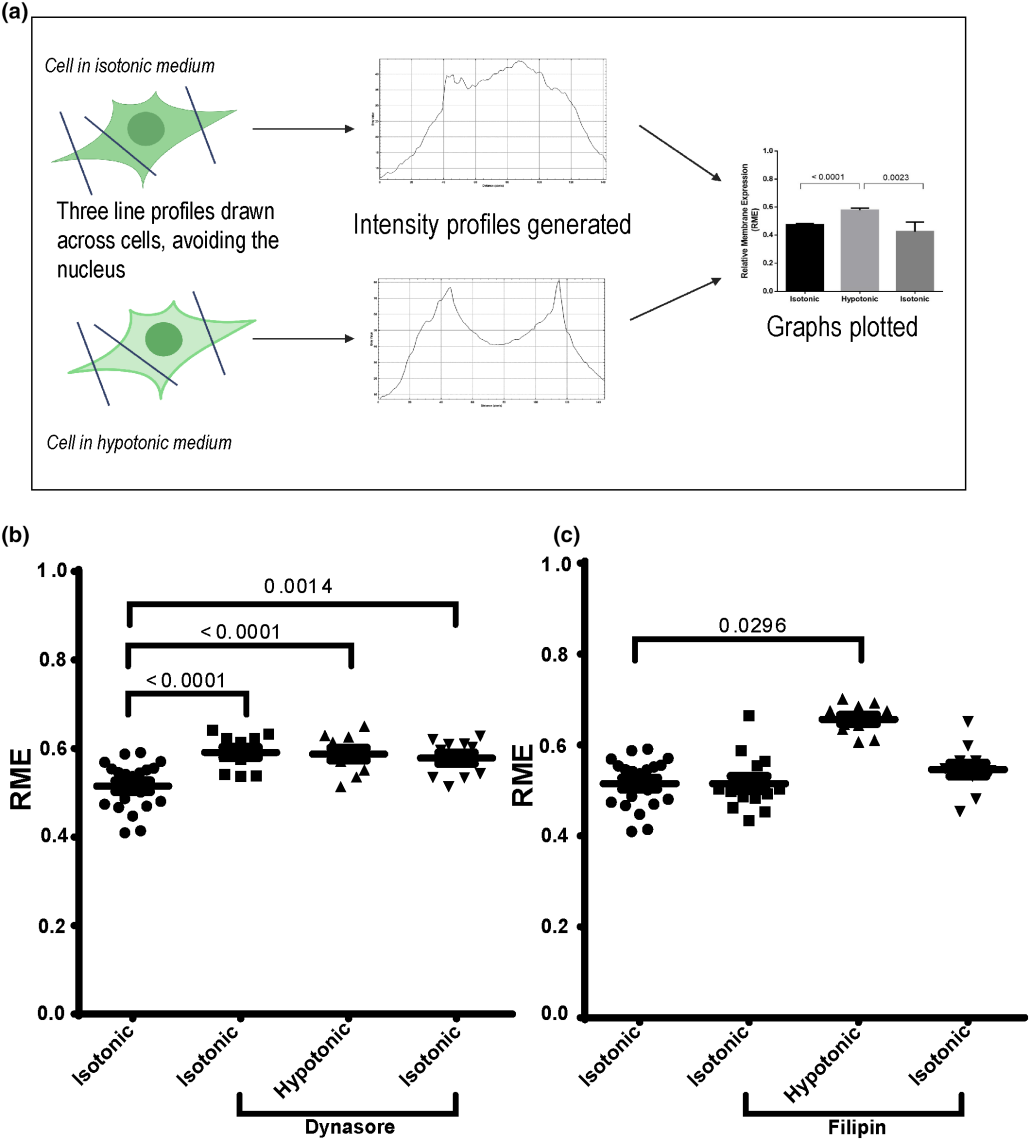
AQP4‐eGFP translocation in HEK293 cells following dynasore and filipin treatment. HEK293 cells were transfected with AQP4‐eGFP and incubated in the presence of filipin and dynasore as described in the methods. AQP4‐eGFP localisation was imaged by live fluorescence microscopy in an isotonic solution at 30 s, at 5 min following exposure to a hypotonic medium, and at 5 min following return back to isotonic medium. (a) representative schematic of how HEK293 transfected with AQP4‐eGFP were analysed to determine relative membrane expression (RME). (b) RME quantification of AQP4‐eGFP in control and dynasore‐treated cells. (c) RME quantification of AQP4‐eGFP in control and filipin‐treated cells. Mean RME for AQP4‐eGFP averaged over three profiles/cell of at least 10 cells per condition in each repeat in three experimental repeats, *n* = 3. For each repeat, *p* values are from one‐way analysis of variance followed by Bonferroni's correction. All data are presented as mean ± SEM.

Transfected HEK293 cells are an appropriate model to study AQP4 translocation (Kitchen et al., 2020). Hundred per cent DMEM was the isotonic control, and 25% DMEM was the hypotonic trigger. Following exposure to a hypotonic environment for only a few seconds, AQP4 translocates from intracellular vesicles to the cell surface of HEK293 cells (Kitchen, Day, Taylor, et al., 2015). To investigate AQP4 internalisation mechanisms, we used well‐established endocytosis inhibitors dynasore and filipin. Dynasore is an inhibitor of the GTPase dynamin needed for vesicle pinching off from the donor membrane. Filipin disrupts cholesterol‐rich rafts and the formation of caveolar structures (Schnitzer et al., 1994) and thus inhibits some clathrin‐independent internalisation pathways. The relative membrane expression (RME) of AQP4‐eGFP was measured under isotonic and hypotonic conditions (Figure 1a). In control HEK293 cells pre‐incubated with 10 μM dynasore for 30 min, significantly increased AQP4 localisation was observed at the cell surface under both isotonic and hypotonic conditions, indicating that internalisation of AQP4 is dynamin‐dependent (Figure 1b; Figure S2). When cells were pre‐incubated with 15 μM filipin for 45 min, the RME of AQP4 in isotonic and hypotonic conditions was similar to that of the control cells (Figure 1c; Figure S2) suggesting that AQP4 does not internalise via caveolar structures.

### 3.2 AQP4‐eGFP trafficking dependence on cytoskeleton

**FIGURE 2:**
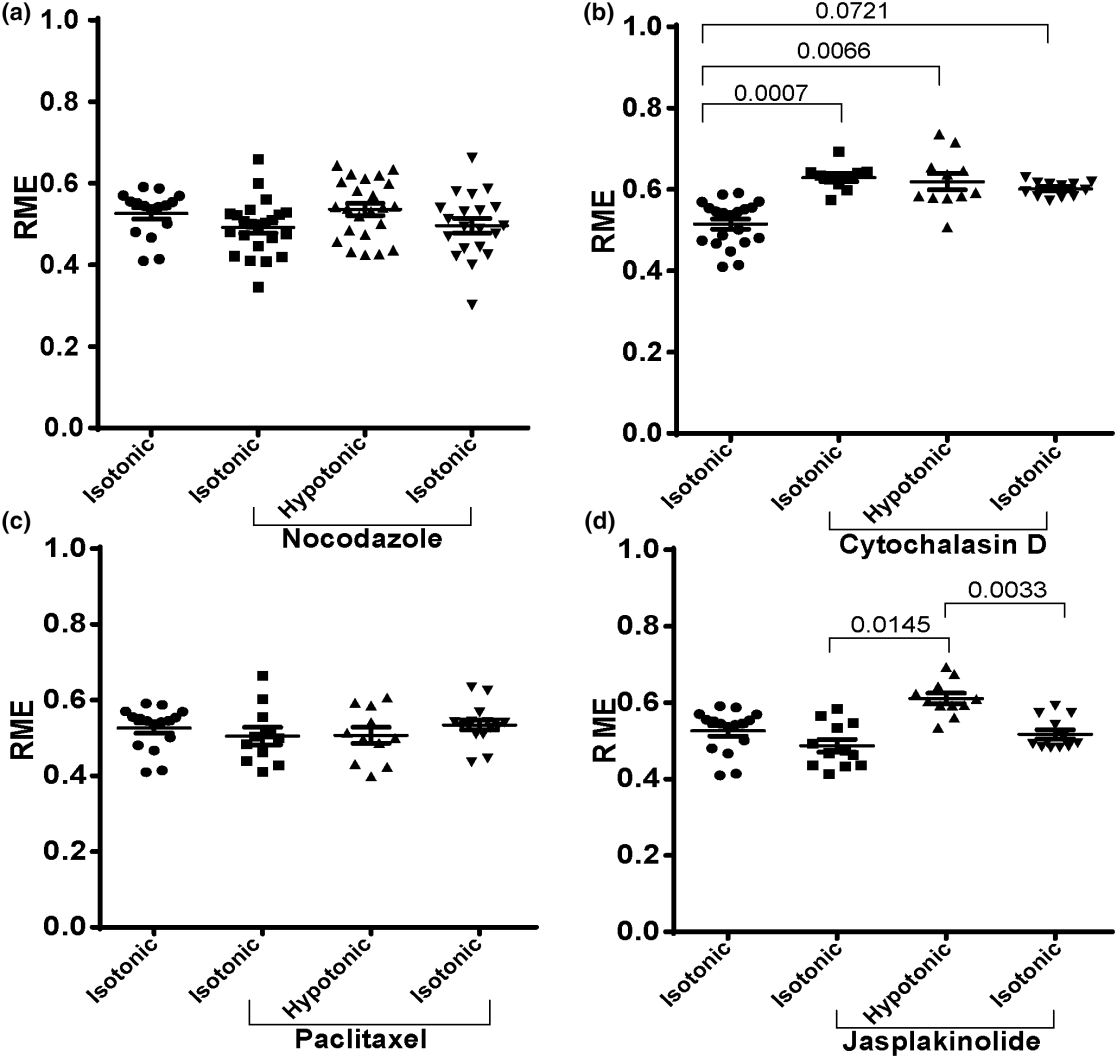
AQP4‐eGFP translocation in HEK293 cells in the presence of cytoskeleton‐modifying drugs. HEK293 cells were transfected with AQP4‐eGFP and incubated in the presence of cytoskeleton modifying drugs as described in the Methods. AQP4‐eGFP localisation was imaged by live fluorescence microscopy in an isotonic solution at 30 s, at 5 min following exposure to a hypotonic medium, and at 5 min following return back to isotonic medium. Quantification of AQP‐eGFP relative membrane expression (RME) in AQP4 + nocodazole (a), AQP4 + cytochalasin D (b), AQP4 + paclitaxel (c) AQP4 + jasplakinolide (d). Mean RME for AQP4‐eGFP averaged over three profiles/cell of at least 10 cells per condition in each repeat in three experimental repeats, *n* = 3. For each repeat, *p* values are from one‐way analysis of variance followed by Bonferroni's correction. All data are presented as mean ± SEM.

Vesicular internalisation and transport across short or long distances often requires cytoskeleton support. AQP4 trafficking was investigated in the presence of cytoskeleton dynamics‐modifying drugs. Cytochalasin D inhibits the elongation phase of actin polymerisation, disrupting the formation of an organised network and leading to the development of filamentous aggregates (Casella et al., 1981). Nocodazole inhibits microtubule elongation, through its interaction with tubulin, and has also been shown to reduce the total number of microtubules in cells (Cassimeris et al., 1986). Jasplakinolide stabilises actin filaments, while paclitaxel stabilises microtubule filaments. Pre‐treatment of transfected HEK293 cells with 30 μM nocodazole for 45 min significantly inhibited AQP4‐eGFP translocation to the cell surface under hypotonic conditions (Figure 2a; Figure S2) suggesting the role of microtubule polymerisation in recycling AQP4‐eGFP‐positive vesicles to the cell surface. Pre‐treatment of transfected HEK293 cells with 5 μM cytochalasin D for 45 min significantly increased AQP4‐eGFP accumulation at the cell surface that did not further increase in the presence of hypotonic conditions. However, returning the cells back to isotonic medium did not cause AQP4‐eGFP internalisation suggesting a role for actin polymerisation in AQP4‐positive vesicle internalisation (Figure 2b; Figure S2). Pre‐treatment of the cells with 50 nM paclitaxel for 20 min had a similar effect to that of nocodazole—inhibition of AQP4‐eGFP translocation to the cell surface under hypotonic conditions (Figure 2c; Figure S2) indicating that microtubule dynamics are important for AQP4‐eGFP‐positive vesicle translocation. Pre‐treatment of the cells with 150 nM jasplakinolide for 30 min did not have any significant effect on AQP4‐eGFP translocation (Figure 2d; Figure S2). These results collectively suggest a role for cytoskeletal dynamics in the translocation of AQP4‐positive vesicles to and from the cell surface.

### 3.3 AQP4‐eGFP trafficking via early and recycling endosomes

**FIGURE 3:**
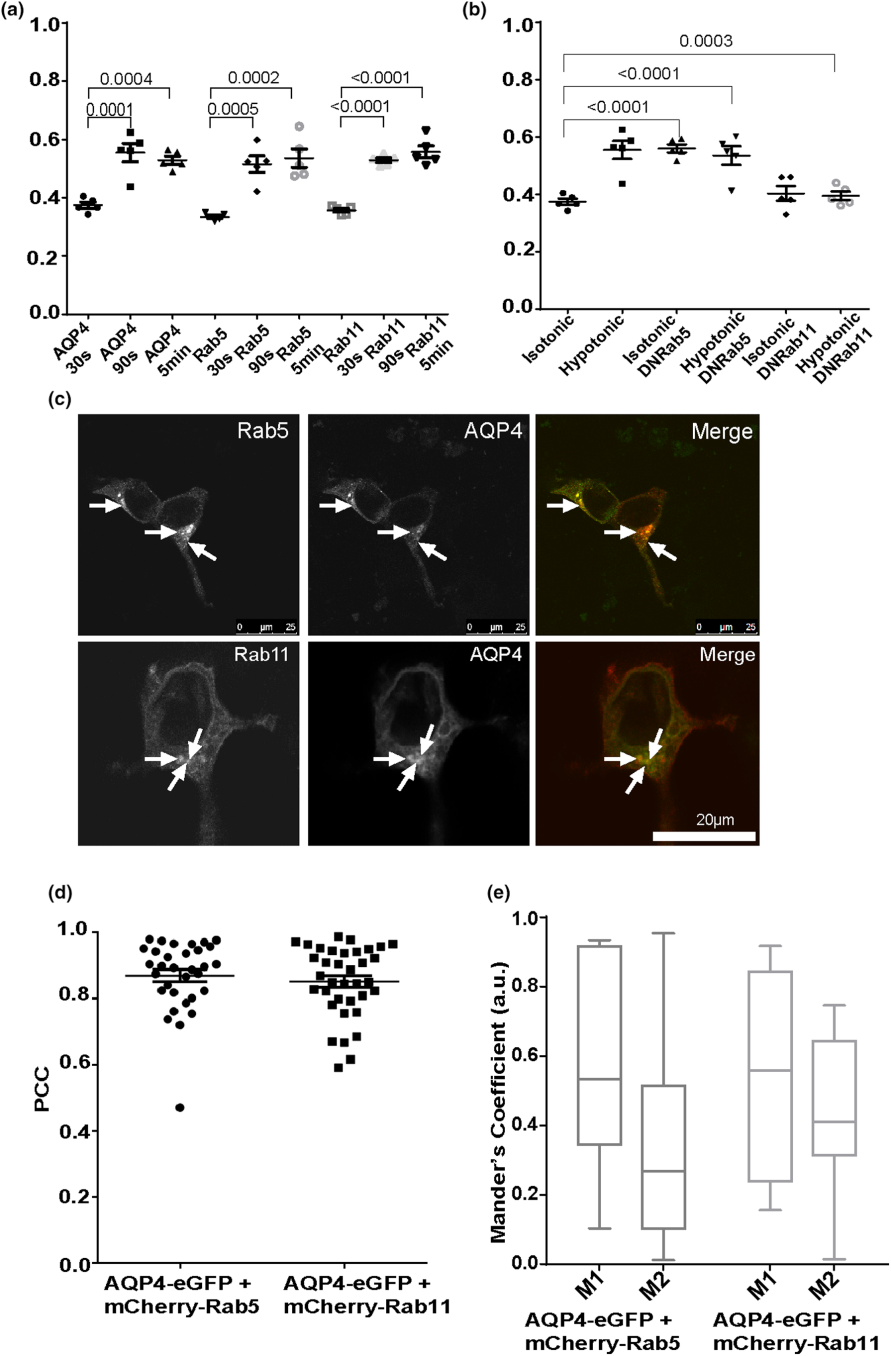
AQP4‐eGFP translocation in HEK293 cells via early and recycling endosomes. HEK293 cells were transfected with AQP4‐eGFP in combination with one of the following: mCherry‐Rab5, mCherry‐Rab11, mCherry‐Rab5DN or DsRed‐Rab11DN. AQP4‐eGFP localisation was imaged by live fluorescence microscopy in an isotonic solution at 30 s, at 5 min following exposure to a hypotonic medium and at 5 min following return back to isotonic medium. Mean RME for AQP4‐eGFP and mCherry‐tagged Rab5 and Rab11 (a), and AQP4eGFP co‐transfected with Rab5DN and Rab11DN (b), averaged over three profiles/cell of at least 10 cells per condition in each repeat in three experimental repeats, *n* = 3. Cells transiently transfected with AQP4‐eGFP (green), mCherry‐Rab5 or mCherry‐Rab11 (red) and the merged images (yellow) (c). Images of each channel were taken sequentially at 63x magnification. The localisation of AQP4‐eGFP and mCherry‐Rab5 overlaps in many areas (white arrows), as does the localisation of AQP4‐eGFP and mCherry‐Rab11 (white arrows). The scale bar is 25 μm. Correlation (PCC) in selected cells (d) and co‐occurrence (e) in selected cells of AQP4‐eGFP with mCherry‐Rab5 and colocalisation of AQP4‐eGP with mCherry‐Rab11. For each repeat, *p* values are from a one‐way analysis of variance followed by Bonferroni's correction. All data are presented as mean ± SEM.

We hypothesised that the most likely route of AQP4 translocation between the cell surface and intracellular environment is via early and recycling endosomes. To explore this, AQP4‐eGFP was co‐transfected in HEK293 cells with either wild‐type or dominant negative (DN) forms of mCherry‐Rab5 and mCherry‐Rab11. DN forms of Rab‐GTPases mimic a loss‐of‐function phenotype and as such are established tools to inhibit vesicular trafficking at specific steps. The RME of AQP4, mCherry‐Rab5 and mCherry‐Rab11 was measured following exposure to hypotonic conditions. The RME of Rab5 and Rab11 followed a similar pattern to that of AQP4 (Figure 3a). The RME of both Rabs at 30 s was approximately 0.4 and 90 s after a hypotonic trigger, the RME of Rab5 and Rab11 rose to approximately 0.6, showing that a larger percentage of both were localised to the cell surface. After 5 min, RME remained at approximately 0.6, which would indicate that the movement of Rab5 and Rab11 occurred mostly within the first 90 s.

To further confirm the role of early and recycling endosomes in AQP4 translocation, the RME of AQP4‐eGFP was measured in cells co‐transfected with either mCherry‐Rab5(DN) or DsRed‐Rab11(DN). In isotonic conditions, in the presence of Rab5(DN), the RME of AQP4‐eGFP was similar to control cells exposed to hypotonic conditions and did not further increase when cells were shifted to hypotonic conditions (Figure 3b). The RME of AQP4‐eGFP when co‐transfected with Rab11(DN) was similar to the control cells exposed to isotonic medium and did not further increase when cells were shifted to hypotonic medium (Figure 3b). These results collectively suggest that impairment of Rab5 and Rab11 function diminished AQP4 translocation in response to changes in tonicity.

To further investigate the role of early and recycling endosomes in AQP4 trafficking, we measured the colocalisation of AQP4 with early and recycling markers Rab5 and Rab11 respectively (Figure 3c). The Pearson's correlation coefficient (PCC) of AQP4‐eGFP and mCherry‐Rab5 was found to be approximately 0.8 (Figure 3d), indicating that the AQP4‐eGFP and mCherry‐Rab5 proteins strongly associate in the same space. AQP4‐eGFP and mCherry‐Rab11 had a PCC of approximately 0.77 (Figure 3d), which indicated a strong association between the two proteins.

Next, the colocalisation was further examined with the calculation of the Manders coefficients, M1 and M2. The M1 value for AQP4‐eGFP and mCherry‐Rab5 was 0.63 and the M2 was 0.55, showing that mCherry‐Rab5 was present on 63% of AQP4‐eGFP‐positive structures while AQP4‐eGFP was present on 55% of the mCherry‐Rab5‐positive vesicles. The M1 value for AQP4‐eGFP and mCherry‐Rab11 was 0.65 and the M2 was 0.50, showing that mCherry‐Rab11 was present on 65% of AQP4‐eGFP‐positive puncta while AQP4‐eGFP was present in 50% of mCherry‐Rab11‐positive vesicles. These results demonstrate that approximately 60% of Rab5‐positive and Rab11‐positive vesicles contain AQP4 and approximately 50% of AQP4 localises with Rab5‐positive and Rab11‐positive vesicles (Figure 3e).

### 3.4 Endogenous AQP4 internalisation in human primary astrocytes

**FIGURE 4:**
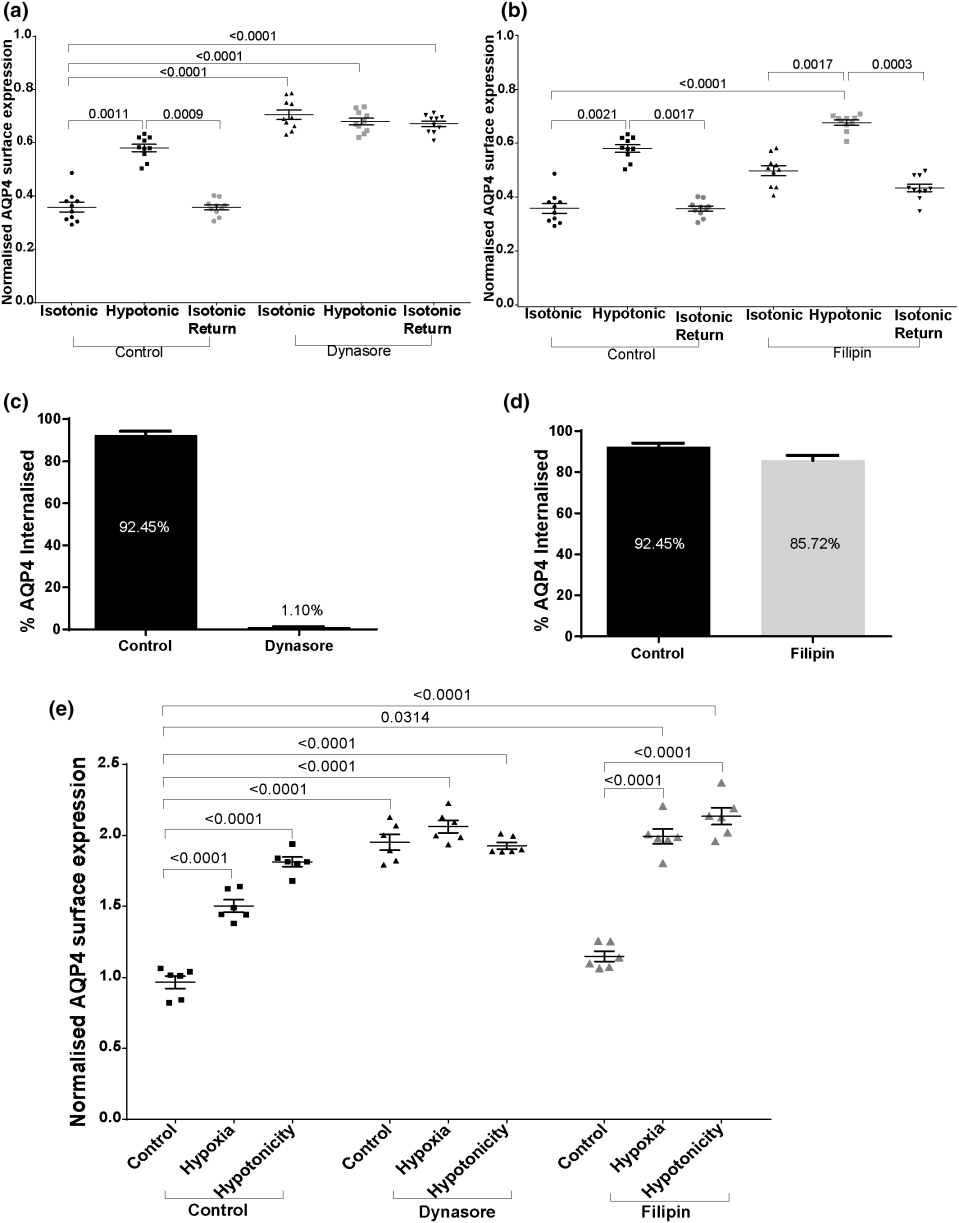
AQP4 internalisation in primary human astrocytes following dynasore and filipin treatment. Surface expression of endogenous AQP4 in primary human astrocytes following treatments was measured by surface biotinylation assay as described in the methods. Treatment with DMSO was used as a vesicle control. (a) Surface expression of AQP4 in control and dynasore‐treated cells in hypotonic and isotonic conditions; (b) surface expression of AQP4 in control and filipin‐treated cells in hypotonic and isotonic conditions; (c) (d) percentage of internalised AQP4 in isotonic and hypotonic media following dynasore and filipin treatment respectively. (e) Surface expression of AQP4 in control, hypoxic and hypotonic conditions following treatment with dynasore and filipin. Control black bar represents normoxic and isotonic conditions; *n* = 3 for each repeat, *p*‐values are from one‐way analysis of variance followed by Tukey's correction. All data are presented as mean ± SEM.

Translocation of endogenous AQP4 in human primary astrocytes in response to either hypotonicity, hypothermia or hypoxia has been observed previously (Kitchen, Day, Salman, et al., 2015; Salman et al., 2017) which suggests that the use of hypotonicity as an in vitro model of cytotoxic oedema is appropriate. To investigate whether similar trafficking mechanisms are true for endogenous AQP4 in a more physiologically relevant model, we performed similar experiments in human primary astrocytes where the surface expression of AQP4 was measured using cell surface biotinylation. Surface expression levels of AQP4 were measured in isotonic conditions, following exposure to hypotonic conditions, and following return to isotonic conditions following pre‐incubation of the cells with either DMSO (Figure S3) or vesicular‐trafficking‐modifying drugs. The same concentrations and incubation times were used as for HEK293 cells. Endogenous AQP4 trafficking in astrocytes was similar to AQP4‐eGFP trafficking in HEK293 cells. Following exposure to hypotonic conditions, AQP4 translocated to the cell surface as indicated by increased surface expression values. This translocation is reversible by returning cells back to isotonic medium (Figure 4a; Figure S3). Incubation of astrocytes in the presence of dynasore significantly increased surface expression of AQP4 that did not further change when cells were shifted to isotonic medium (Figure 4a), suggesting that internalisation of endogenous AQP4 is dynamin‐dependent. Since dynasore has been reported to reduce the amount of labile cholesterol in the plasma membrane (Preta et al., 2015), we also measured AQP4 trafficking following incubation with another dynamin inhibitor dyngo‐4a. Inhibition of dynamin with dyngo‐4a resulted in similar AQP4 uptake inhibition (Figure S4), supporting our earlier findings suggesting AQP4 internalisation is dynamin‐dependent. Incubation of astrocytes with filipin did not affect AQP4 translocation following changes in tonicity, suggesting that AQP4 internalisation in astrocytes is likely to use a clathrin‐dependent mechanism (Figure 4b). Additionally, we used pitstop 2, an inhibitor blocking interaction of clathrin with amphiphysin and observed reduced uptake of AQP4 from the cell surface (Figure S4). The amount of internalised AQP4 was measured by returning the cells to the incubator for 30 min after biotinylation, and then removing the remaining surface biotin moieties using a reducing agent to reduce the S‐S bond in the biotinylation reagent (Figure 4c,d). Similar effects of drugs on AQP4 translocation were observed between hypotonic and hypoxic conditions (Figure 4e), confirming that hypotonicity is appropriate to model the hypoxic conditions relevant to brain trauma when studying AQP4 translocation mechanisms.

### 3.5 Effect of cytoskeleton‐modifying drugs on endogenous AQP4 trafficking in human primary astrocytes

**FIGURE 5:**
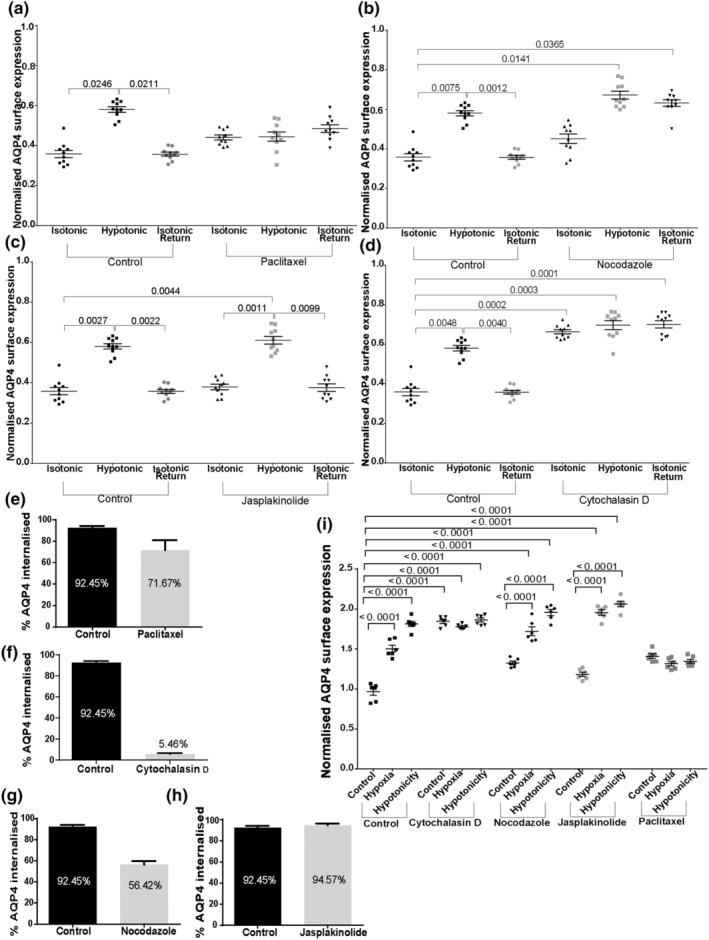
AQP4 translocation in primary human astrocytes in response to hypoxia and hypotonicity following treatment with cytoskeleton‐modifying drugs. Surface expression of endogenous AQP4 in primary human astrocytes following exposure to hypotonic medium and return back to isotonic medium was measured by surface biotinylation assay as described in the methods following pre‐incubation with paclitaxel (a), nocodazole (b), jasplakinolide (c) and cytochalasin D (d). Treatment with DMSO was used as a vesicle control. (e–h) percentage of internalised AQP4 following exposure to hypotonic medium and pre‐treatments with paclitaxel, nocodazole, jasplakinolide and cytochalasin D respectively. I, surface expression of endogenous AQP4 in primary human astrocytes following exposure to hypotonic medium or hypoxia measured by surface biotinylation assay *n* = 3 for each repeat, *p*‐values are from one‐way analysis of variance followed by Tukey's correction. All data are presented as mean ± SEM.

Endogenous AQP4 translocation was measured following changes in tonicity and pre‐incubation with cytoskeleton dynamics‐modifying drugs. Pre‐incubation of human primary astrocytes with paclitaxel resulted in inhibition of AQP4 translocation to the cell surface following cell exposure to hypotonic medium (Figure 5a). Nocodazole treatment did not affect AQP4 translocation to the cell surface but blocked the internalisation of AQP4 following return of cells from hypotonic to isotonic medium (Figure 5b). These results suggest a role for microtubule dynamics in AQP4‐positive vesicle trafficking. While jasplakinolide did not have an effect on AQP4 trafficking (Figure 5c), pre‐incubation of the cells with cytochalasin D resulted in increased surface localisation of AQP4 in isotonic conditions that did not further change by shifting cells to hypotonic medium (Figure 5d), suggesting a role for actin cytoskeleton dynamics in AQP4 internalisation. These results were further supported by quantifying AQP4 internalisation (Figure 5e–h). Although we routinely use extracellular hypotonicity to induce AQP4 trafficking, the large osmotic gradient required is probably not physiologically relevant, even for conditions such as cytotoxic oedema that involve rapid transmembrane water flux through AQP4. Therefore, to more realistically model cytotoxic oedema following stroke or traumatic brain injury/spinal cord injury, the above experiments were repeated using hypoxia (1% O_2_ and 5% CO_2_) for 48 h as the trigger. The AQP4 translocation appears to follow the same pattern in response to both hypotonic and hypoxic conditions when quantified in the presence of cytoskeleton dynamics‐modifying drugs (Figure 5i), further validating the hypotonicity model.

## 4 DISCUSSION

The translocation of AQP4 from intracellular vesicles to the cell surface occurs when a hypoxic (or hypotonic) environment triggers an influx of calcium ions, through transient receptor potential vanilloid 4 (TRPV4) channels, which bind to and activate CaM (Kitchen et al., 2020). Activated CaM activates an adenylyl cyclase, which in turn leads to cyclic adenosine monophosphate (cAMP) production that further activates PKA which phosphorylates AQP4 (Kitchen, Day, Salman, et al., 2015). The results presented here begin to elucidate the mechanism by which AQP4 relocalises to the cell surface through vesicular trafficking following phosphorylation as well as its internalisation mechanisms. Membrane protein trafficking mechanisms are complex, requiring several key regulators and key steps in order to successfully sort and deliver the cargo to its correct destination. AQP trafficking pathways differ among family members, with each depending on different triggers and different mechanisms for movement to and from the cell surface (Conner et al., 2013). Although a few AQPs have well‐described trafficking mechanisms, such as AQP1 and AQP2, the regulation of these is not the same as that of AQP4.

### 4.1 AQP4 internalisation routes

Several drugs were used to investigate AQP4 internalisation mechanisms, dynamin inhibitors dynasore and dyngo‐4a, filipin, an inhibitor that binds to free cholesterol and affects cholesterol rafts, and pitstop 2, an inhibitor interrupting clathrin–amphiphysin interaction. The results with dynasore and filipin were validated in two different cell systems (HEK293 and primary human astrocytes) and two AQP4 relocalisation triggers (hypotonicity and hypoxia). Inhibition of dynamin with dynasore resulted in increased localisation of AQP4 at the cell surface in both hypotonic and hypoxic conditions and in both cell systems, that did not further change when cells were returned to isotonic medium. Inhibition of dynamin with dyngo‐4a in astrocytes supported the dynasore data. The results highlight two important points: First, AQP4 appears to continuously cycle between vesicles and the cell surface, as dynamin inhibition in isotonic conditions increases its surface expression levels (Figures 1b, 4a; Figure S4) by blocking AQP4 internalisation and thus shifting the balance of cycling towards cell surface accumulation. Second, the role of dynamin in vesicle pinching has been associated with both clathrin‐dependent endocytosis (CME) and clathrin‐independent endocytosis (CIE) and our data suggest that AQP4 internalisation occurs in a dynamin‐dependent manner. As filipin did not interfere with AQP4 trafficking (Figures 1c, 4b,e), AQP4 uptake is less likely to happen via the CIE route; however, we cannot exclude all CIE routes, as filipin only interferes with internalisation mechanisms depending on cholesterol‐rich rafts. Additionally, pitstop 2 data in astrocytes does not allow us to fully distinguish between CME and CEI as although pitstop 2 specifically inhibits interaction of amphiphysin with the amino terminal domain of clathrin and thus blocks CME, there is evidence that pitstop 2 also inhibits CIE (Dutta et al., 2012). Recently, caveolin‐1 knockout was found to reduce AQP4 expression in a mouse stroke model, although it is not clear whether this was due to a direct role of caveolin‐1 in AQP4 internalisation, or a secondary effect on AQP4 (Filchenko et al., 2020). Like AQP4, AQP2 is also internalised through CME and has been shown to colocalise with clathrin in clathrin‐coated pits (Noda & Sasaki, 2005; Sun et al., 2002). The clathrin AP2‐adaptor complex has also been shown to interact with AQP4 (Madrid et al., 2001). More recently, it was shown that the internalisation of AQP4 can be regulated by clathrin interacting with dynamin, due to the increase in AQP4 cell surface localisation when chlorpromazine was used to inhibit the formation of clathrin‐coated pits (Tham et al., 2016). However, it has also been shown that AQP2 internalisation may occur through association with caveolin‐1, as both were observed in caveolar structures in transfected Madin‐Darby canine kidney (MDCK) cells (Aoki et al., 2012). AQP2 has been shown to associate with membrane rafts and cholesterol depletion resulted in delay of AQP2 exit from the trans‐Golgi network and AQP2 accumulation in the plasma membrane and inhibition of AQP2 internalisation (Procino et al., 2010), further supporting a role for lipid rafts in AQP2 translocation. In CME, and CIE involving caveolae, dynamin is a key mediator of internalisation. It has been shown in a number of studies that AQP2 is dependent on dynamin for endocytosis, both by inhibition of dynamin through the use of small molecules, or with dominant negative forms of the GTPase (Dollerup et al., 2015; Sun et al., 2002). Our results agree with published data showing dynamin involvement in AQP4 internalisation (Tham et al., 2016). Comparison and detailed dissection of AQP4 internalisation mechanisms in future may reveal additional cell‐dependent differences in AQP4 endocytosis.

### 4.2 AQP4 trafficking via early and recycling endosomes

The two Rabs, Rab5 and Rab11, were chosen as the most likely candidates to sustain AQP4 trafficking. Following internalisation, most membrane protein cargo is targeted to Rab5‐positive early or sorting endosomes where the destination of the cargo is determined by sorting into different sub‐compartments. Two major routes for cargo exiting early endosomes are either degradation via late endosomes and lysosomes or recycling back to the cell surface either directly or via specialised Rab11‐positive recycling endosomes. Colocalisation of other AQPs with Rab5‐ and Rab11‐positive endosomes has been reported previously (Takata et al., 2008). Our results support the hypothesis for the role of early and recycling endosomes in AQP4 trafficking by several lines of evidence. Firstly, there was a significant colocalisation observed between AQP4 and Rab5/Rab11 in HEK293 cells (Figure 3c–e), suggesting a role for Rab5‐positive early endosomes and Rab11‐positive recycling endosomes in AQP4 trafficking. Secondly, Rab5‐positive and Rab‐11‐positive endosomes demonstrated a similar pattern of localisation to AQP4 in response to hypotonic conditions (Figure 3a) suggesting co‐trafficking. Thirdly, when cells were transfected with dominant negative mutants of Rab5 and Rab11, AQP4 translocation was impaired. The presence of a Rab5(DN) mutant resulted in a significant increase in AQP4 localised to the cell surface, compared to the control. Like dynamin inhibition, Rab5 inhibition also resulted in AQP4 accumulation at the cell surface by blocking AQP4 internalisation and shift in the recycling equilibrium (Figure 3b). In the presence of Rab11(DN), the basal level of AQP4 present at the cell surface was unchanged, but shifting to hypotonic medium, no longer increased cell surface localisation of AQP4, indicating a block in AQP4 recycling to the cell surface (Figure 3b). Similarities in AQP2 and AQP4 trafficking can be noted following the internalisation step. Once the vesicle containing AQP2 is internalised, it is localised to a Rab5‐positive early endosome (Wang et al., 2020; Wong et al., 2020). Following the sorting that occurs in the early endosome, AQP2 is targeted to the recycling endosome, where Rab5 is replaced by Rab11 (Sonnichsen et al., 2000). This conversion has not been studied extensively but has been shown to be required for recycling to occur (Haas et al., 2005; Mani et al., 2016; Sun et al., 2012). It has been shown previously that AQP2 colocalises with Rab11 in vesicles. Our colocalisation data show that more than 50% of AQP4 is present on both Rab5‐ and Rab11‐ positive endosomes, suggesting that a small fraction of AQP4 colocalises with both Rab5 and Rab11. This agrees with a previous report demonstrating that both Rabs contribute to compartmentalisation within the same continuous membrane resulting in a fraction of endosomes that are positive for both Rab proteins (Sonnichsen et al., 2000).

### 4.3 Role of cytoskeletal dynamics in vesicular trafficking of AQP4

The role of the cytoskeleton in vesicular trafficking in eukaryotes has been widely described, but it is important to note that the data are specific to the cell, organism and cargo type under investigation (Granger et al., 2014).

The role of actin cytoskeleton dynamics in AQP4 trafficking was investigated using cytochalasin D, which inhibits the elongation phase of actin polymerisation, disrupting the formation of an organised network and leading to the development of filamentous aggregates (Casella et al., 1981) and jasplakinolide, a compound that stabilises actin filaments. While stabilisation of actin filaments with jasplakinolide did not affect AQP4 translocation in response to hypotonicity or hypoxia (Figures 2d and 5c,h,i), inhibition of actin polymerisation with cytochalasin D increased AQP4 accumulation at the cell surface in isotonic medium that did not further change when cells were shifted to either hypotonic of hypoxic conditions (Figures 2b and 5d,f,i). These results indicate that actin polymerisation is required for AQP4 internalisation, and that inhibition with cytochalasin D affects continuous AQP4 cycling by shifting the equilibrium towards the cell surface and restricting internalisation into endosomes. The actin cytoskeleton and its remodelling are an integral part of the trafficking machinery of the cell (Lanzetti, 2007). More specifically, with regard to AQP2, actin has been shown to directly bind to the water channel (Noda et al., 2004). This complex has been suggested to be integral to the targeting of AQP2 to the apical membrane of the kidney collecting duct (Noda et al., 2008). It has been shown previously in rat and mouse astrocytes that actin plays a role in AQP4 localisation in astrocytes (Nicchia et al., 2008). Actin and AQP4 colocalise with each other, and the use of cytochalasin D to alter actin polymerisation subsequently disrupts AQP4 cell surface localisation (Nicchia et al., 2008). It should also be noted that in rat astrocytes, cytochalasin D was reported to increase water permeability (Nicchia et al., 2008), which is consistent with the increase in AQP4 cell surface expression that we observed in isotonic medium. Although the actin cytoskeleton was proposed to play a role in AQP2 internalisation, it has also been shown that inhibiting actin polymerisation with both cytochalasin D and latrunculin B did not affect AQP2 trafficking (Tajika et al., 2005). However, a more recent study proposed that the conversion of F‐actin to G‐actin allowed AQP2‐positive vesicles to be internalised (Wang et al., 2017), showing that in fact AQP2 endocytosis can be affected by the actin cytoskeleton structure. Finally, apart from the involvement of the actin cytoskeleton in internalisation of AQP2, the movement of AQP2‐positive vesicles are also dependent on actin cytoskeleton dynamics. It was first proposed that the actin cytoskeleton, and more specifically the F‐actin state, was required to depolymerise to allow for the movement of AQP2 to the plasma membrane (Noda et al., 2008). AQP2 is in fact the trigger for this depolymerisation, which is a consequence of the AQP2 movement (Yui et al., 2012). Our results suggest that unlike for AQP2, the actin cytoskeleton, and perhaps more specifically F‐actin, is necessary for AQP4 internalisation.

The role of microtubules in trafficking has been assessed, with recent studies showing their involvement not only in positioning of organelles, but also their role in cargo transport to the plasma membrane in a quick and targeted manner (Fourriere et al., 2020). The potential role of microtubules in the trafficking of AQP4 has been reported previously, with a suggestion that the disruption of the network impedes this movement. When moving from the cytosol to the cell surface, vesicles containing GFP‐tagged AQP4 were shown to move along cytoskeletal structures. When nocodazole was used, this movement along this trajectory was disrupted (Mazzaferri et al., 2013). The role of microtubule network dynamics was investigated by using nocodazole, that inhibits microtubule elongation and paclitaxel that stabilises microtubule filaments. Inhibition of microtubule dynamics resulted in the accumulation of AQP4 in intracellular vesicles and restricted AQP4 translocation to the cell surface in response to hypotonicity in HEK293 cells (Figure 2a,c). These data are in agreement with previous experiments showing the role of microtubules in the movement of AQP4 from the cytosol to the plasma membrane in MDCK cells (Mazzaferri et al., 2013). In human primary astrocytes, the basal level of AQP4 cell surface expression was not affected by pre‐incubation of the cells with nocodazole when compared to the isotonic control, and under hypotonic conditions, AQP4 was able to relocalise to the cell surface. However, when isotonicity was restored, AQP4 was not internalised, perhaps suggesting that there are different underlying mechanisms for internalisation of AQP4 for basal recycling and internalisation following trigger‐induced relocalisation. The differences in AQP4 translocation following nocodazole treatment in HEK293 cells and primary astrocytes indicate that the requirements for AQP4 vesicle trafficking may be different between the cell types. AQP4 relocalisation was dependent on microtubule dynamics in HEK293 cells, whereas only AQP4 internalisation following trigger‐induced relocalisation was dependent on microtubule dynamics in primary astrocytes (Figures 2a,c and 5a,b). Microtubules have been shown to be an important component of AQP2 trafficking. They have been associated with both the internalisation of the water channel as well as targeting to the cell surface (reviewed in (Centrone et al., 2022)).

## 5 CONCLUSIONS

**FIGURE 6:**
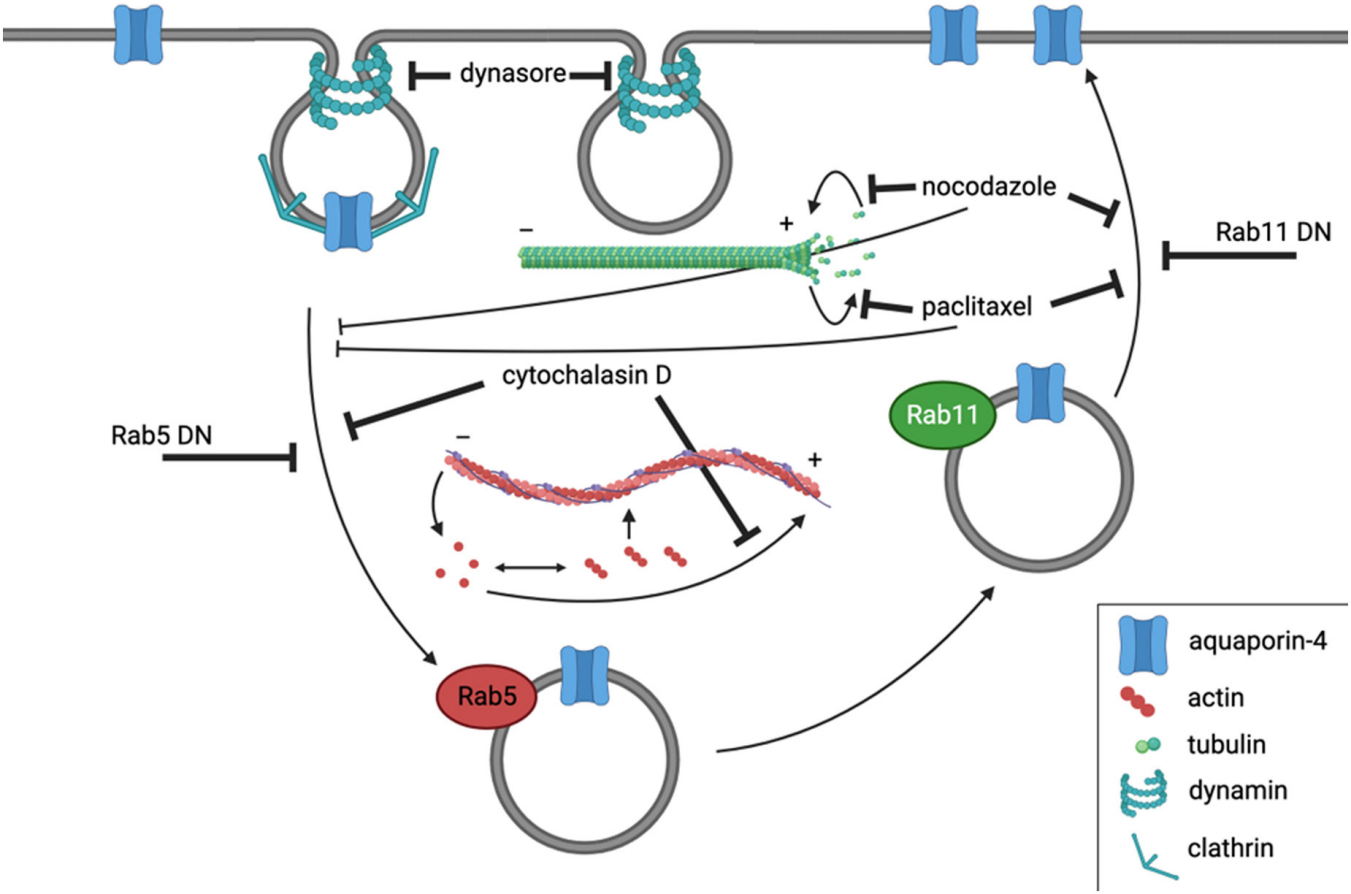
Molecular mechanisms of AQP4 translocation in mammalian cells. AQP4 continuously cycles between the cell surface, Rab5‐positive early and Rab11‐positive recycling endosomes in mammalian cells. AQP4 internalisation is dynamin‐dependent and AQP4 translocation mechanisms are impaired upon inhibition of Rab5 and Rab11 function as well as cytoskeleton dynamics using cytoskeleton dynamics‐modifying drugs. Created with biorender.com.

The reversibility of cell surface translocation in tandem with use of various pharmacological agents has allowed us to propose that under physiological conditions, at least some AQP4 continuously cycles between the cell surface and intracellular vesicles and that this cycling can be shifted towards recycling or internalisation depending on which step of vesicular trafficking is blocked (Figure 6). Collectively, our data show that AQP4 trafficking in primary human astrocytes happens in a similar manner to that in HEK293 cells with the exception of the role of microtubule dynamics, and largely confirms that HEK293 cells are an appropriate model for use when investigating AQP4 trafficking. Endogenous AQP4 in astrocytes behaved in a similar way to the transiently transfected AQP4‐eGFP in HEK293 cells. The use of hypotonicity has also been shown to be an appropriate trigger for studying AQP4 subcellular relocalisation in vitro (Kitchen et al., 2020), with no major differences when compared to hypoxia. Understanding AQP4 vesicular translocation mechanisms at the molecular level brings us one step closer to finding a therapeutic agent to reduce AQP4 cell surface localisation and hence membrane water permeability. This gives hope to the millions of CNS oedema patients worldwide, for whom there is no preventative therapy.

## AUTHOR CONTRIBUTIONS

**Andrea Markou:** Formal analysis; investigation; writing – original draft; writing – review and editing. **Philip Kitchen:** Conceptualization; investigation; methodology; writing – review and editing. **Ahmed Aldabbagh:** Formal analysis; investigation. **Mariaelena Repici:** Methodology; visualization. **Mootaz M. Salman:** Conceptualization; writing – review and editing. **Roslyn M. Bill:** Conceptualization; formal analysis; supervision; writing – review and editing. **Zita Balklava:** Conceptualization; formal analysis; investigation; supervision; writing – original draft; writing – review and editing.

## CONFLICT OF INTEREST STATEMENT

R.M.B., P.K. and M.M.S. are shareholders in Estuar Pharmaceuticals.

### PEER REVIEW

The peer review history for this article is available at https://www.webofscience.com/api/gateway/wos/peer‐review/10.1111/jnc.16029.

## Supporting information


Data S1.

## Data Availability

The data that support the findings of this study are available from the corresponding author upon reasonable request.
