# Supplementary material for: Mechanisms of aquaporin‐4 vesicular trafficking in mammalian cells
Source: J Neurochem. 2023 Dec 16;168(2):100–14. doi: 10.1111/jnc.16029 (PMC10953025; doi:10.1111/jnc.16029)
Supplement: Supplementary file 1 — Data S1. [file JNC-168-100-s001.pdf]

SUPPLEMENTARY MATERIAL FOR:

Mechanisms of aquaporin-4 vesicular trafficking in mammalian cells.

Andrea Markou[1,2], Philip Kitchen[1], Ahmed Aldabbagh[1], Mariaelena Repici[1], Mootaz M Salman[3,4], Roslyn M Bill[1] and Zita Balklava[1].*

[1]College of Health and Life Sciences, Aston University, Aston Triangle, Birmingham B4 7ET, UK;
[2]School of Biosciences, Faculty of Health and Medical Sciences, University of Surrey, Stag Hill, University Campus, Guildford GU2 7XH, UK;
[3]Department of Physiology, Anatomy and Genetics, University of Oxford, Oxford OX1 3QX, UK.
[4]Kavli Institute for NanoScience Discovery, University of Oxford, Oxford OX1 3QU, UK.

*Correspondence z.balklava@aston.ac.uk

All experimental work was done at Aston University.

Author information:
Andrea Markou; a.markou@surrey.ac.uk; ORCID ID: 0000-0003-3912-5823;
Philip Kitchen; p.kitchen1@aston.ac.uk; ORCID ID: 0000-0002-1558-4673;
Ahmed Aldabbagh; 190236771@aston.ac.uk;
Mariaelena Repici; m.repici@aston.ac.uk; ORCID ID: 0000-0002-9420-528X;
Mootaz M Salman; mootaz.salman@dpag.ox.ac.uk; ORCID ID: 0000-0002-5683-1706; ISN membership nr 23393;
Roslyn M Bill; R.M.Bill@aston.ac.uk; ORCID ID: 0000-0003-1331-0852;
Zita Balklava; z.balklava@aston.ac.uk; ORCID ID: 0000-0001-9039-9710;

Supplementary tables

Supplementary table 1. P values for graph 1B; F (3, 40) = 27.99.

P Values	Isotonic Control	Hypotonic Control	Isotonic Return Control	Isotonic Dynasore Treated	Hypotonic Dynasore Treated	Isotonic Return Dynasore Treated
Isotonic Control		< 0.0001	0.4830	< 0.0001	< 0.0001	0.0014
Hypotonic Control	< 0.0001		0.0023	0.9611	0.9899	0.9990
Isotonic Return Control	0.4830	0.0023		0.0074	0.0002	0.0055
Isotonic Dynasore Treated	< 0.0001	0.9611	0.0074		0.9998	0.9709
Hypotonic Dynasore Treated	< 0.0001	0.9899	0.0002	0.9998		0.9611
Isotonic Return Dynasore Treated	0.0014	0.9990	0.0055	0.9709	0.9969	

Supplementary table 2. P values for graph 1C; F (3, 53) = 20.37.

P Values	Isotonic Control	Hypotonic Control	Isotonic Return Control	Isotonic Filipin Treated	Hypotonic Filipin Treated	Isotonic Return Filipin Treated
Isotonic Control		< 0.0001	0.4830	0.3413	< 0.0001	0.5377
Hypotonic Control	< 0.0001		0.0023	0.6302	0.1869	0.9803
Isotonic Return Control	0.4830	0.0023		0.2432	0.0005	0.2980
Isotonic Filipin Treated	0.3413	0.6302	0.2432		0.0296	0.9997
Hypotonic Filipin Treated	< 0.0001	0.1869	0.0005	0.0296		0.3123
Isotonic Return Filipin Treated	0.5377	0.9803	0.2980	0.9997	0.3123	

Supplementary table 3. P values for graph 2A; F (3, 78) = 2.123.

P Values	Isotonic Control	Hypotonic Control	Isotonic Return Control	Isotonic Nocodazole Treated	Hypotonic Nocodazole Treated	Isotonic Return Nocodazole Treated
Isotonic Control		< 0.0001	0.4830	0.9609	0.0691	0.9153
Hypotonic Control	< 0.0001		0.0023	0.0004	0.0281	0.0016
Isotonic Return Control	0.4830	0.0023		0.7599	0.2844	0.7140
Isotonic Nocodazole Treated	0.9609	0.0004	0.7599		0.2279	>0.9999
Hypotonic Nocodazole Treated	0.0691	0.0281	0.2844	0.2279		0.3755
Isotonic Return Nocodazole Treated	0.9153	0.0016	0.7140	>0.9999	0.3755	

Supplementary table 4. P values for graph 2B; F (3, 49) = 18.46.

P Values	Isotonic Control	Hypotonic Control	Isotonic Return Control	Isotonic Cytochalasin D Treated	Hypotonic Cytochalasin D Treated	Isotonic Return Cytochalasin D Treated
Isotonic Control		< 0.0001	0.4830	<0.0001	<0.0001	0.0005
Hypotonic Control	< 0.0001		0.0023	0.4167	0.9035	0.9972
Isotonic Return Control	0.4830	0.0023		0.0009	0.0046	0.0237
Isotonic Cytochalasin D Treated	<0.0001	0.4167	0.0009		0.9845	0.9466
Hypotonic Cytochalasin D Treated	<0.0001	0.9035	0.0046	0.9845		0.9996
Isotonic Return Cytochalasin D Treated	0.0005	0.9972	0.0237	0.9466	0.9996	

Supplementary table 5. P values for graph 2C; F (3, 49) = 0.6862.

P Values	Isotonic Control	Hypotonic Control	Isotonic Return Control	Isotonic Paclitaxel Treated	Hypotonic Paclitaxel Treated	Isotonic Return Paclitaxel Treated
Isotonic Control		< 0.0001	0.4830	0.3506	0.3662	0.1127
Hypotonic Control	< 0.0001		0.0023	0.0396	0.0479	0.0258
Isotonic Return Control	0.4830	0.0023		0.2514	0.2653	0.1937
Isotonic Paclitaxel Treated	0.3506	0.0396	0.2514		0.9570	0.9591
Hypotonic Paclitaxel Treated	0.3662	0.0479	0.2653	0.9570		>0.9999
Isotonic Return Paclitaxel Treated	0.1127	0.0258	0.1937	0.9591	>0.9999	

Supplementary table 6. P values for graph 2D; F (3, 47) = 12.11.

P Values	Isotonic Control	Hypotonic Control	Isotonic Return Control	Isotonic Jasplakinolide Treated	Hypotonic Jasplakinolide Treated	Isotonic Return Jasplakinolide Treated
Isotonic Control		< 0.0001	0.4830	0.9638	0.0002	0.6363
Hypotonic Control	< 0.0001		0.0023	0.0599	0.8711	0.4664
Isotonic Return Control	0.4830	0.0023		0.6473	0.0036	0.3638
Isotonic Jasplakinolide Treated	0.9638	0.0599	0.6473		0.0145	0.9858
Hypotonic Jasplakinolide Treated	0.0002	0.8711	0.0036	0.0145		0.0033
Isotonic Return Jasplakinolide Treated	0.6363	0.4664	0.3638	0.9858	0.0033	

Supplementary table 7. P values for graph 3A; F (8, 36) = 20.83.

P Values	AQP4 30s	AQP4 90s	AQP4 5 mins	Rab5 30s	Rab5 90s	Rab5 5 mins	Rab11 30s	Rab11 90s	Rab11 5 mins
AQP4 30s		0.0001	0.0004	0.8962	0.0006	<0.0001	0.9995	0.0001	<0.0001
AQP4 90s	0.0001		0.9903	<0.0001	0.8950	0.9989	<0.0001	0.9906	>0.9999
AQP4 5 mins	0.0004	0.9903		<0.0001	>0.9999	>0.9999	<0.0001	>0.9999	0.9835
Rab5 30s	0.8962	<0.0001	<0.0001		0.0005	0.0002	0.9968	<0.0001	<0.0001
Rab5 90s	0.0006	0.8950	>0.9999	0.0005		0.9982	<0.0001	>0.9999	0.8624
Rab5 5 mins	<0.0001	0.9989	>0.9999	0.0002	0.9982		<0.0001	>0.9999	0.9976
Rab11 30s	0.9995	<0.0001	<0.0001	0.9968	<0.0001	<0.0001		<0.0001	<0.0001
Rab11 90s	0.0001	0.9906	>0.9999	<0.0001	>0.9999	>0.9999	<0.0001		0.9840
Rab11 5 mins	<0.0001	>0.9999	0.9835	<0.0001	0.8624	0.9976	<0.0001	0.9840	

Supplementary table 8. P values for graph 3C; $F(5, 24) = 14.42$.

P Values	Isotonic	Hypotonic	Isotonic DN Rab5	Hypotonic DN Rab5	Isotonic DN Rab11	Hypotonic DN Rab11
Isotonic		0.0002	0.0001	0.0007	0.9468	0.9880
Hypotonic	0.0002		>0.9999	0.9900	0.0014	0.0007
Isotonic DN Rab5	0.0001	>0.9999		0.9733	0.0009	0.0005
Hypotonic DN Rab5	0.0007	0.9900	0.9733		0.0059	0.0031
Isotonic DN Rab11	0.9468	0.0014	0.0009	0.0059		0.9998
Hypotonic DN Rab11	0.9880	0.0007	0.0005	0.0031	0.9998	

Supplementary table 9. P values for graph 4A; F (5, 54) = 129.3.

P Values	Isotonic Control	Hypotonic Control	Isotonic Return Control	Isotonic Dynasore Treated	Hypotonic Dynasore Treated	Isotonic Return Dynasore Treated
Isotonic Control		0.0011	0.9992	< 0.0001	< 0.0001	< 0.0001
Hypotonic Control	0.0011		0.0009	0.0503	0.1395	0.0791
Isotonic Return Control	0.9992	0.0009		< 0.0001	< 0.0001	< 0.0001
Isotonic Dynasore Treated	< 0.0001	0.0503	< 0.0001		0.9868	0.9997
Hypotonic Dynasore Treated	< 0.0001	0.1395	< 0.0001	0.9868		0.9991
Isotonic Return Dynasore Treated	< 0.0001	0.0791	< 0.0001	0.9997	0.9991	

Supplementary table 10. P values for graph 4B; $F (5, 54) = 78.75$.

P Values	Isotonic Control	Hypotonic Control	Isotonic Return Control	Isotonic Filipin Treated	Hypotonic Filipin Treated	Isotonic Return Filipin Treated
Isotonic Control		0.0021	0.9995	0.2923	< 0.0001	0.2819
Hypotonic Control	0.0021		0.0017	0.0745	0.0882	0.0068
Isotonic Return Control	0.9995	0.0017		0.0712	<0.0001	0.1800
Isotonic Filipin Treated	0.2923	0.0745	0.0712		0.0017	0.7124
Hypotonic Filipin Treated	< 0.0001	0.0882	<0.0001	0.0017		0.0003
Isotonic Return Filipin Treated	0.2819	0.0068	0.1800	0.7124	0.0003	

Supplementary table 11. P values for 5A; $F (5, 54) = 25.97$.

P Values	Isotonic Control	Hypotonic Control	Isotonic Return Control	Isotonic Paclitaxel Treated	Hypotonic Paclitaxel Treated	Isotonic Return Paclitaxel Treated
Isotonic Control		0.0246	> 0.9999	0.6610	0.8948	0.4888
Hypotonic Control	0.0246		0.0211	0.0828	0.3750	0.1349
Isotonic Return Control	> 0.9999	0.0211		0.5425	0.8059	0.3821
Isotonic Paclitaxel Treated	0.6610	0.0828	0.5425		0.9962	0.9996
Hypotonic Paclitaxel Treated	0.8948	0.3750	0.8059	0.9962		0.9660
Isotonic Return Paclitaxel Treated	0.4888	0.1349	0.3821	0.9996	0.9660	

Supplementary table 12. P values for graph 5B; $F(5, 54) = 62.47$.

P Values	Isotonic Control	Hypotonic Control	Isotonic Return Control	Isotonic Nocodazole Treated	Hypotonic Nocodazole Treated	Isotonic Return Nocodazole Treated
Isotonic Control		0.0075	> 0.9999	0.8607	0.0141	0.0365
Hypotonic Control	0.0075		0.0012	0.2300	0.9683	> 0.9999
Isotonic Return Control	> 0.9999	0.0012		0.7861	0.0079	0.0712
Isotonic Nocodazole Treated	0.8607	0.2300	0.7861		0.0689	0.1757
Hypotonic Nocodazole Treated	0.0141	0.9683	0.0079	0.0689		0.9902
Isotonic Return Nocodazole Treated	0.0365	> 0.9999	0.0712	0.1757	0.9902	

Supplementary table 13. P values for graph 5C; $F (5, 54) = 56.19$.

P Values	Isotonic Control	Hypotonic Control	Isotonic Return Control	Isotonic Jasplakinolide Treated	Hypotonic Jasplakinolide Treated	Isotonic Return Jasplakinolide Treated
Isotonic Control		0.0027	0.9996	0.9656	0.0044	0.9861
Hypotonic Control	0.0027		0.0022	0.0010	0.9851	0.0008
Isotonic Return Control	0.9996	0.0022		0.8810	< 0.0001	0.9311
Isotonic Jasplakinolide Treated	0.9656	0.0010	0.8810		0.0011	> 0.9999
Hypotonic Jasplakinolide Treated	0.0044	0.9851	< 0.0001	0.0011		0.0099
Isotonic Return Jasplakinolide Treated	0.9861	0.0008	0.9311	> 0.9999	0.0099	

Supplementary table 14. P values for graph 5D; F (5, 54) = 103.1.

P Values	Isotonic Control	Hypotonic Control	Isotonic Return Control	Isotonic Cytochalasin D Treated	Hypotonic Cytochalasin D Treated	Isotonic Return Cytochalasin D Treated
Isotonic Control		0.0048	0.9997	0.0002	0.0003	0.0001
Hypotonic Control	0.0048		0.0040	0.6365	0.7899	0.3772
Isotonic Return Control	0.9997	0.0040		< 0.0001	< 0.0001	< 0.0001
Isotonic Cytochalasin D Treated	0.0002	0.6365	< 0.0001		0.9997	0.9961
Hypotonic Cytochalasin D Treated	0.0003	0.7899	< 0.0001	0.9997		0.9698
Isotonic Return Cytochalasin D Treated	0.0001	0.3772	< 0.0001	0.9961	0.9698	

A B C

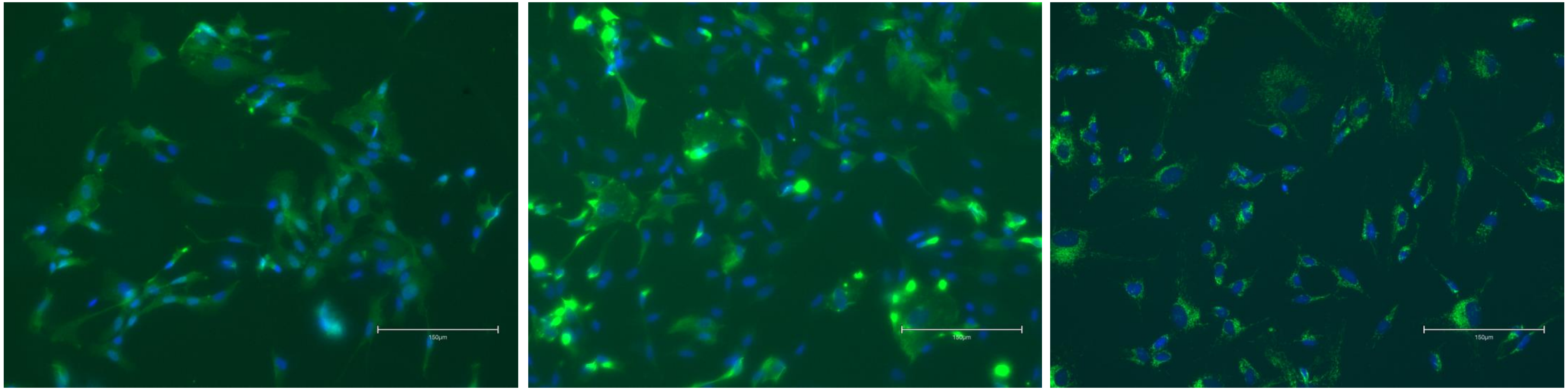

Image	Primary antibody	Secondary antibody
A	Anti-EAAT1 (cell signaling EAAT1 (5684S) / (1:100)/ Rabbit)	Alexa Flour 488 goat anti-rabbit IgG (Invitrogen, A-11008/ 1:1000)
B	AntiGFAP (abcam/ ab68428/ 1:250/ Rabbit)	Alexa Flour 488 goat anti-rabbit IgG (Invitrogen, A-11008/ 1:1000)
C	Anti-S100B (Merck/ ab128906/ 1:50/ Mouse)	Alexa Flour 488 goat anti-mouse IgG (Invitrogen, A-11001/ 1:1000)

Supplementary Figure 1. Confirmation of human primary astrocyte identity with astrocyte- specific markers.
Representative confocal microscopy images of immunocytochemistry staining of human primary astrocytes with astrocyte-specific markers EAAT1 (A), GFAP (B) and S100β (C). Representative confocal images taken at 20x magnification; scale bar 150μm. Antibody information shown for each image in (D).

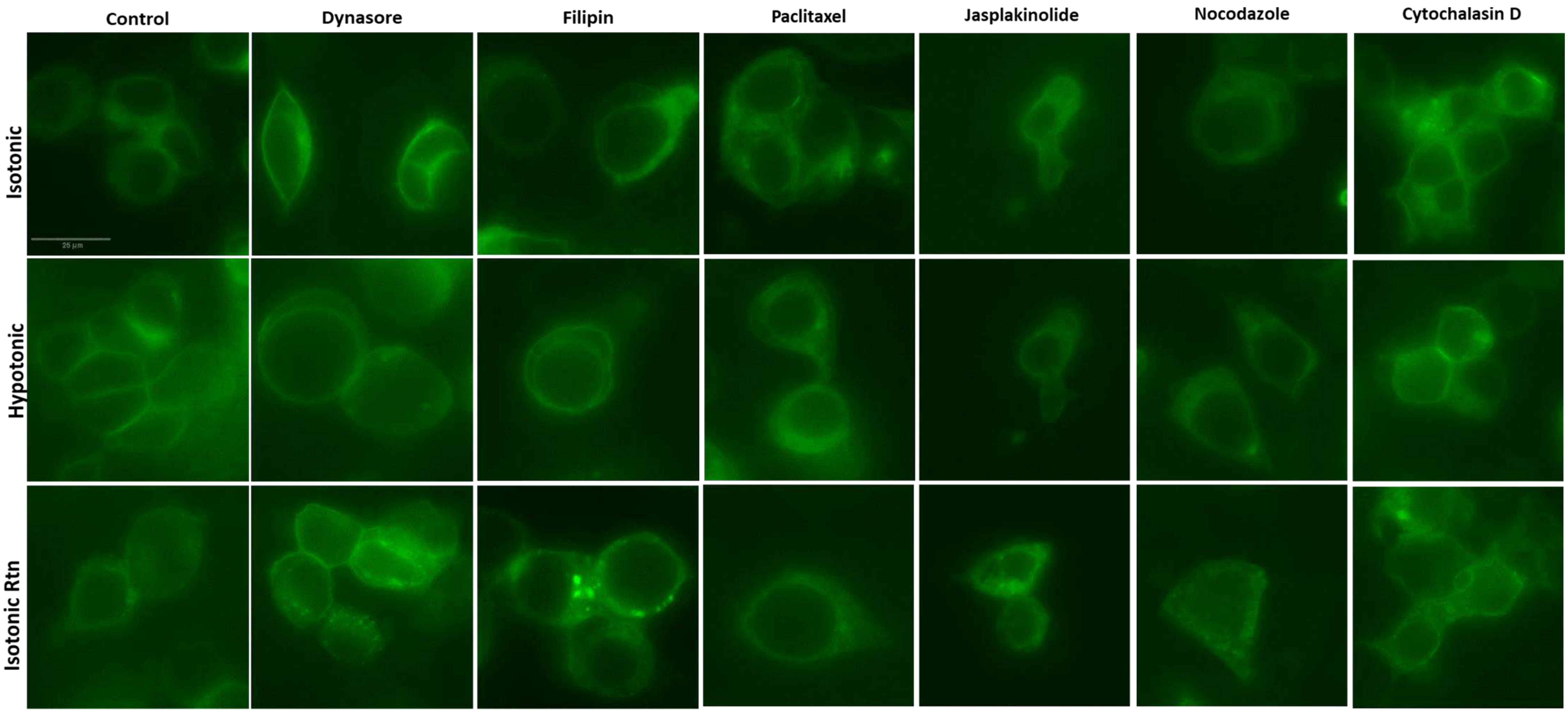

Supplementary Figure 2. AQP4-eGFP localization following treatment with vesicular trafficking modifying drugs. Representative images of HEK293 cells transfected with AQP4-eGFP and treated with various drugs as described in the methods. AQP4-eGFP localisation was imaged using live fluorescence microscopy at three time points: following 30 second exposure in an isotonic medium, following 5 minute exposure in a hypotonic medium and following a 5 minute return back to isotonic medium (Rtn). Magnification 63x, scale bar 25µm.

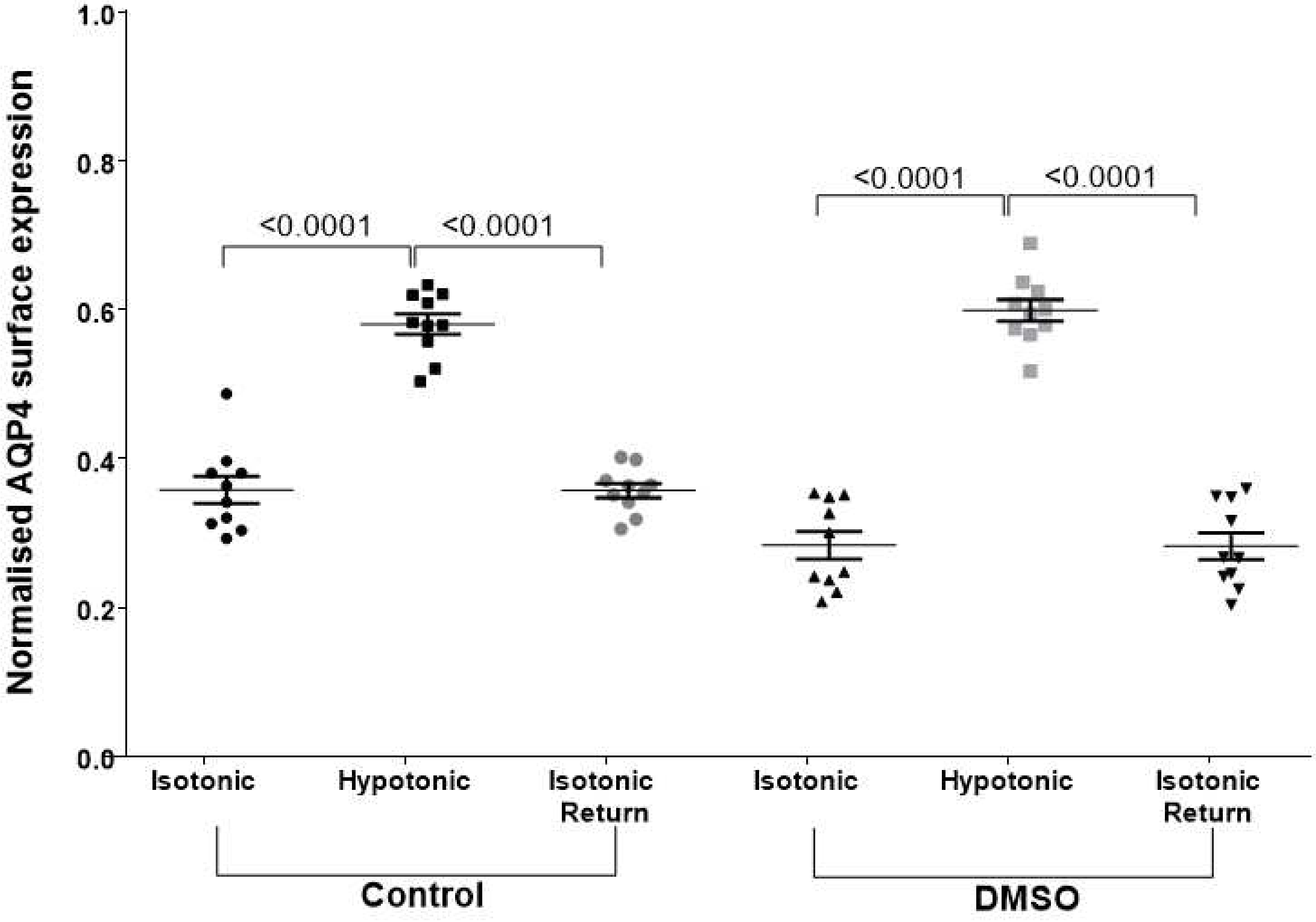

Supplementary Figure 3. Hypotonicity stimulates AQP4-eGFP relocalisation to the cell surface in a reversible manner. Transiently transfected HEK293 cells with AQP4-eGFP were treated with 0.1% DMSO for 45 minutes. This treatment does not disrupt subcellular relocalisation of AQP4 following a hypotonic trigger, and translocation can be reversed when isotonicity is restored. *n* = 3 for each repeat, *p*-values are from one-way analysis of variance followed by Tukey's correction. All data are presented as mean ± S.E.M.

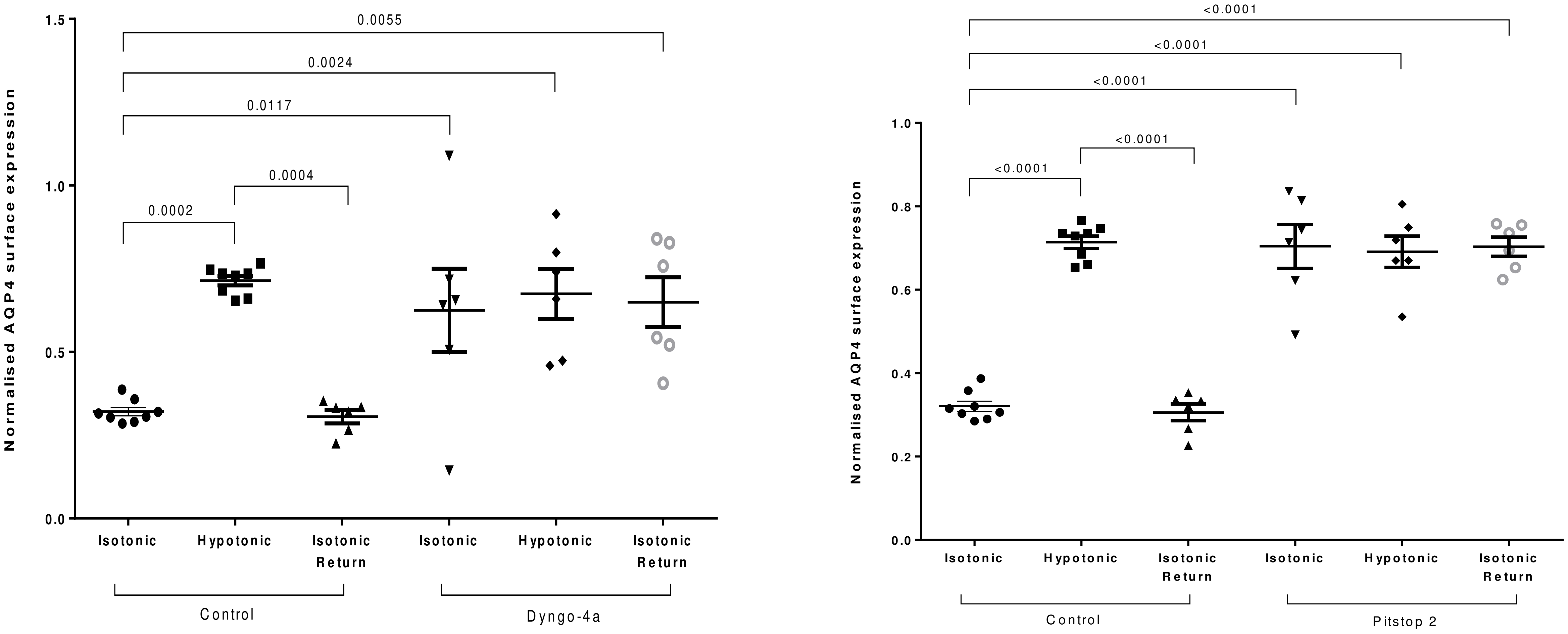

Supplementary Figure 4. AQP4 internalisation in primary human astrocytes following dyngo-4a and pitstop 2 treatment. Surface expression of endogenous AQP4 in primary human astrocytes following treatments was measured by surface biotinylation assay as described in the methods. Treatment with DMSO was used as a vesicle control. **A,** surface expression of AQP4 in control and dyngo-4a treated cells in hypotonic and isotonic conditions; **B,** surface expression of AQP4 in control and pitstop 2 treated cells in hypotonic and isotonic conditions; $n = 3$ for each repeat, *p*-values are from one-way analysis of variance followed by Tukey's correction. All data are presented as mean ± S.E.M.
